# Expression of Concern: MUC1 gene silencing inhibits proliferation, invasion and migration while promoting apoptosis of oral squamous cell carcinoma cells

**DOI:** 10.1042/BSR-20182193_EOC

**Published:** 2020-06-24

**Keywords:** Oral squamous cell carcinoma, MUC1 gene silencing, Proliferation, Invasion, Migration, Apoptosis

The Editorial Office has been made aware of potential issues surrounding the scientific validity of this paper, hence has issued an expression of concern to notify readers whilst the Editorial Office investigates.

